# Etiology, microbiological isolates, and antibiotic susceptibilities in culture-proven pediatric endophthalmitis: a 9-year review

**DOI:** 10.1007/s00417-020-04866-7

**Published:** 2020-08-18

**Authors:** Yao Yang, Lixia Lin, Yujie Li, Zhaoxin Jiang, Cheng Li, Manli Liu, Fang Duan, Xiaofeng Lin

**Affiliations:** State Key Laboratory of Ophthalmology, Zhongshan Ophthalmic Center, Sun Yat-sen University, 54 South Xianlie Road, Guangzhou, 510060 China

**Keywords:** Child, Endophthalmitis, Microbiological profile, Antibiotics, Epidemiology

## Abstract

**Purpose:**

To analyze the etiology, microbiological isolates, and antibiotic susceptibilities of endophthalmitis in pediatric patients.

**Methods:**

Patients aged < 18 years with culture-positive endophthalmitis in Zhongshan Ophthalmic Center between January 2010 and December 2018 were included retrospectively.

**Results:**

A total of 127 patients (127 eyes) were included, and 108 (85%) had posttraumatic endophthalmitis. *Streptococcus* (21.4%), coagulase-negative *Staphylococcus* (14.5%), *Aspergillus* (6.9%), and *Bacillus cereus* (5.3%) were the common organisms. The proportion of *Streptococcus* decreased with age (40.0% in 0–3 years, 16.3% in 4–12 years, and 6.3% in 13–17 years), while coagulase-negative *Staphylococcus* increased from 5.7% to 18.8%. Overall, fluoroquinolones achieved the highest antibiotic susceptibility rate (> 95%), while the susceptibility of isolated bacteria to tobramycin and cefazolin was only 60.2% and 59.4%, respectively. The susceptibility rates of Gram-positive cocci to cephalosporins were nearly 90%. For Gram-negative bacilli, susceptibility to neomycin was 91.3%.

**Conclusion:**

Trauma was the main etiology for pediatric endophthalmitis. Although *Streptococcus* was the most prevalent organism in general, the dominant pathogen varied with age, which merits clinical attention. Fluoroquinolones showed the highest antibiotic efficacy; however, commonly used antibiotics tobramycin and cefazolin showed relatively low antibiotic susceptibility. Thus, antibiotic resistance in pediatric populations merits clinical attention.

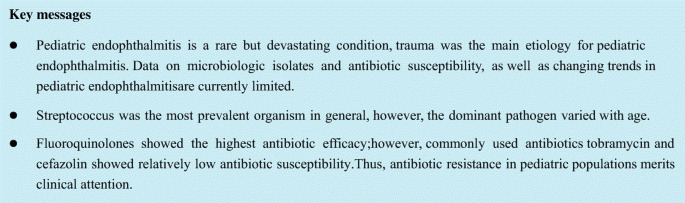



## Introduction

Pediatric endophthalmitis is a rare but devastating condition, resulting in visual impairment and even blindness. The reported incidence of pediatric endophthalmitis following cataract surgery ranges from 0.38 to 0.45% [1, 2]. The incidence is estimated at 2.8–54.2% in the pediatric age group with ocular trauma, which varies by country [3–5]. Children endogenous endophthalmitis is rare; it constitutes only 0.1–4% of all cases of endogenous endophthalmitis [6]. Although the clinical features and microbiological profile of pediatric endophthalmitis are different from those of adult endophthalmitis [3, 7], few studies have focused on the pediatric form.

Recent reports on pediatric endophthalmitis mostly focused on endophthalmitis due to a specific etiology such as pediatric cataract surgery [8], trauma [3, 9], and endogenous infection [6] or focused on a specific type of endophthalmitis caused by a certain pathogen [10]. Only a few studies focused on pediatric endophthalmitis from all causes. Among these, the sample sizes in most studies were small, with an even smaller number culture-positive cases, thereby providing limited information for clinical practice [11–13]. Zhang and colleagues reported the etiology and microorganism spectrum of 271 pediatric endophthalmitis cases in eastern China, of which 147 cases were culture positive; yet antibiotic susceptibility was not provided [14]. Information on antibiotic susceptibilities of endophthalmitis in children is critical for guiding empiric antibiotic treatment. Data on microbiologic isolates and antibiotic susceptibility, as well as changing trends in pediatric endophthalmitis, could help guide empiric antibiotic treatment but are currently limited.

In this study, we reviewed a comparatively large sample of 127 inpatient, culture-proven cases of pediatric endophthalmitis, with the following aims: (1) to describe the distribution of etiology, microbiological isolates, and antibiotic susceptibilities in pediatric patients with endophthalmitis and (2) to identify the changing trends in microbiological profile in pediatric endophthalmitis. Our findings provide information to ophthalmologists for the empiric treatment of pediatric endophthalmitis.

## Methods

This study was performed in compliance with the principles of the Declaration of Helsinki and was approved by the Institutional Ethics Committee of Zhongshan Ophthalmic Center, Sun Yat-sen University. The requirement for patient consent was waived given the retrospective nature of the study.

### Study population

We retrospectively collected data of 127 consecutive cases of culture-positive pediatric endophthalmitis in Zhongshan Ophthalmic Center, southern China, from January 2010 to December 2018. Patients ≥ 18 years old, without positive culture outcomes, or without culture were excluded.

### Procedures

Epidemiological data including patient age and sex were collected. Based on the patient’s medical history, symptoms, and signs, the etiology of endophthalmitis was classified and recorded by a senior ophthalmologist during hospitalization. All endophthalmitis cases were then divided into four endophthalmitis groups: posttraumatic, postoperative, endogenous, and others (including infections associated with keratitis and corneal-suture removal). Patients were also divided into three age groups: 0–3 years, 4–12 years, and 13–17 years. The 9-year study period was divided into 2010–2014 and 2015–2018 to analyze microbiological profile trends.

Aqueous/vitreous taps for culture were performed during surgery under general anesthesia. Aqueous humor from the anterior chamber was aspirated through the corneal limbus with a needle on a 1-mL syringe. Vitreous specimens were collected through the pars plana prior to antibiotic injection or vitrectomy, using a needle or vitrector. Samples were then inoculated in trypticase soy broth (BACT/ALERT® SA and BACT/ALERT® SN, BioMerieux, Inc., Marcy-l’Étoile, France) overnight at 37 °C. Subsequently, the broth was inoculated onto sheep blood agar and potato glucose agar for the growth of bacterial cultures and fungal cultures, respectively. [15–17]. All bacterial isolates were subjected to species identification on an automated microbiological system Vitek 2 Compact (BioMerieux, Inc., Marcy-l’Étoile, France); all fungi isolates were identified by experienced technicians according to fungal morphology. Antibiotic susceptibility testing of isolated bacteria was performed using both the Kirby-Bauer disc diffusion method and minimal inhibitory concentration (MIC) methods, according to different antibiotics. The antibiotic susceptibility was determined in accordance with the methods of the Clinical and Laboratory Standards Institute (CLSI). Bacterial susceptibilities were recorded as “sensitive,” “intermediate,” or “resistant.” For the purpose of this study, being “sensitive” and being “intermediate” were both considered sensitive. The following antibiotics were used for susceptibility tests: fluoroquinolones (moxifloxacin, levofloxacin, ofloxacin), cephalosporins (ceftazidime, cefazolin, and cefuroxime), aminoglycosides (tobramycin and neomycin), penicillin, vancomycin, and chloramphenicol. There are very few results for vancomycin and moxifloxacin, as routine tests were started in 2017. It was not until 2016 that we began routine penicillin susceptibility testing.

### Statistical analysis

All analyses were performed using SPSS version 16.0 (SPSS Inc., Chicago, IL, USA). The results of all culture-positive specimens were analyzed. The culture results and susceptibility data are presented as categorical variables and expressed as percentages. Differences between groups were compared using the chi-square test. *P* < 0.05 was considered significant.

## Results

During the 9-year study period, 127 of 459 pediatric patients (< 18 years) with clinically diagnosed endophthalmitis had positive cultures, and all infections were monocular. The mean age of patients was 6.5 ± 4.0 years (range, 8 months to 17 years), and the proportion of male to female was 2.4:1 (90:37). With respect to age distribution, patients in the 4–12-year-old group accounted for the largest proportion (60.6%, *n* = 77), followed by those in the 0–3 years group (26.8%, *n* = 34) (Table 1).

**Table 1:** Distribution of endophthalmitis by sex and age group

	Total *n* = 127	Post-traumatic *N* = 108	Endogenous *N* = 12	Post-operative *N* = 4	Others *N* = 3
Sex					
Male	90 (70.9%)	77 (71.3%)	8 (66.7%)	4 (100%)	1 (33.3%)
Female	37 (29.1%)	31 (28.7%)	4 (33.3%)	0	2 (66.7%)
Age (years)					
0–3	34 (26.8%)	30 (27.8%)	2 (16.7%)	0	2 (66.7%)
4–12	77 (60.6%)	67 (62.0%)	5 (41.7%)	4 (100%)	1 (33.3%)
13–17	16 (12.6%)	11 (10.2%)	5 (41.7%)	0	0

Based on etiology, 108 (85.0%) endophthalmitis cases occurred after eye trauma. There were 95 and 13 cases of penetrating and rupture injury, respectively; injuries caused by metal wire/nail (18.5%) were the most common cause, followed by plants (17.6%), and scissors (13.0%). Twelve (9.4%) patients were diagnosed as having endogenous endophthalmitis; four (33.3%) of them had potential systemic risk factors for endophthalmitis: one with prior upper respiratory tract infection and fever; one with acute sinusitis; one had recurrent pneumonia and arthritis before onset; and one received systemic steroid therapy. In four (3.1%) patients, endophthalmitis occurred after intraocular surgery, of which two were associated with trabeculectomy and the remaining two occurred after penetrating keratoplasty and Ahmed glaucoma valve implantation, respectively. The group of other endophthalmitis included three (2.4%) patients with infectious keratitis (*n* = 2) and after corneal suture removal (*n* = 1).

The microbiological profile is shown in Table 2. Of the 127 cases, 4 were polymicrobial infections; therefore, in effect, the total number of isolates was 131. Of these, 61.1% were Gram-positive bacteria including 48.1% Gram-positive cocci and 13.0% Gram-positive *bacillus*, 22.9% were Gram-negative *bacillus*, and 16.0% were fungal isolates. No Gram-negative cocci were isolated. All isolates in the postoperative endophthalmitis group were bacterial, while fungal isolates accounted for 50.0% of all endogenous endophthalmitis cases.

**Table 2:** Microbiological profile of endophthalmitis in children

	Total isolates *N* = 131	Isolates by percentage of each endophthalmitis category			
		Posttraumatic *N* = 112	Endogenous *N* = 12	Postoperative *N* = 4	Others *N* = 3
Gram-positive cocci	63 (48.1%)	50.0%	16.6%	50.0%	100%
*Streptococcus*.	28 (21.4%)	22.3%	0	25.0%	66.7%
Coagulase-negative *Staphylococcus*	19 (14.5%)	14.3%	8.3%	25.0%	33.3%
*Staphylococcus epidermidis*	6 (4.6%)	5.4%	0	0	0
*Granulicatella*	3 (2.3%)	2.7%	0	0	0
*Kocuria* spp.	3 (2.3%)	2.7%	0	0	0
Other Gram-positive cocci	10 (7.6%)	8.0%	8.3%	0	0
Gram-positive bacilli	17 (13.0%)	15.2%	0	0	0
*Bacillus cereus*	7 (5.3%)	6.3%	0	0	0
*Bacillus subtilis*	6 (4.6%)	5.4%	0	0	0
Other *Bacillus* spp.	2 (1.5%)	1.8%	0	0	0
Other Gram-negative bacilli	2 (1.5%)	1.8%	0	0	0
Gram-negative bacilli	30 (22.9%)	21.4%	33.3%	50.0%	0
*Pseudomonas* spp.	7 (5.3%)	2.7%	25%	25.0%	0
*Pseudomonas aeruginosa*	6 (4.6%)	1.8%	25%	25.0%	0
*Aeromonas*	4 (3.1%)	3.5%	0	0	0
*Enterobacter cloacae*	3 (2.3%)	2.7%	0	0	0
*Escherichia coli*	2 (1.5%)	1.8%	0	0	0
*Xanthomonas*	2 (1.5%)	0.9%	8.3%	0	0
*Serratia* spp.	2 (1.5%)	1.8%	0	0	0
Other Gram-negative bacilli	10 (7.6%)	8.0%	0	25.0%	0
Fungus	21 (16.0%)	13.4%	50.0%	0	0
*Aspergillus* spp.	9 (6.9%)	7.1%	8.3%	0	0
*Mucor*	5 (3.8%)	1.8%	16.6%	0	0
*Fusarium* spp.	2 (1.5%)	0.9%	8.3%	0	0
*Penicillium* sp.	2 (1.5%)	0.9%	8.3%	0	0
Uncertain^a^	3 (2.3%)	2.7%	8.3%	0	0
Total	131 (100%)	100%	100%	100%	100%

^a^Uncertain fungus include fungus that could not be identified for a specific species

We analyzed the changing trends of microbiological profile between the periods of 2010–2014 and 2015–2018. Overall, the proportion of Gram-positive bacterial infection increased. Gram-positive cocci increased from 43.4% to 53.5% (*P* = 0.25), and Gram-positive *bacillus* increased from 9.2% to 17.9% (*P* = 0.13). The proportion of Gram-negative *bacillus* infections decreased from 26.3% to 17.9% (*P* = 0.25), and that of fungal infections decreased from 21.1% to 10.7% (*P* = 0.12).

*Streptococcus* (21.4%) was the most prevalent organism in our study, followed by coagulase-negative staphylococcus (14.5%), of which *Staphylococcus epidermidis* accounted for 4.6% infections. *Bacillus cereus* (5.3%) and *Bacillus subtilis* (4.6%) were the most common Gram-positive bacilli. *Pseudomonas aeruginosa* (4.6%) accounted for the highest rate of Gram-negative *bacillus* infections. The most prevalent isolated fungus was *Aspergillus* (6.9%). The distribution of isolated organisms in different age groups is shown in Table 3. *Streptococcus* was the most prevalent isolates in the 0–3-year-old age group and accounted for 40% infections; it decreased to 16.3% in 4–12-year-old group, slightly lower than coagulase-negative staphylococcus (17.5%). It further decreased to 6.3% in the 13–17-year-old group, in whom coagulase-negative staphylococci were the predominant isolates. The proportion of fungal infection was 11.4% in the 0–3-year-old group and increased with age, reaching 31.2% in the 13–17-year-old group.

**Table 3:** Distribution of microbiological profile in different age groups

	Isolates by percentage of age groups (years)		
	0–3 *N* = 35	4–12 *N* = 80	13–17 *N* = 16
Gram-positive cocci	54.3%	46.3%	43.8%
*Streptococcus*	40.0%	16.3%	6.3%
Coagulase-negative *Staphylococcus*	5.7%	17.5%	18.8%
*Staphylococcus epidermidis*	6.5%	6.3%	0
*Granulicatella*	5.7%	1.3%	0
*Kocuria* spp.	0	3.8%	0
Other Gram-positive cocci	2.8%	7.5%	18.8%
Gram-positive bacilli	17.1%	13.8%	0
*Bacillus cereus*	5.7%	6.3%	0
*Bacillus subtilis*	5.7%	5.0%	0
Other bacilli	2.8%	1.3%	0
Other Gram-negative bacilli	2.8%	1.3%	0
Gram-negative bacillus	17.1%	25.0%	25.0%
*Pseudomonas* spp.	0	7.5%	6.3%
*Pseudomonas aeruginosa*	0	6.3%	6.3%
*Aeromonas*	2.8%	2.5%	6.3%
Enteric bacilli	0	3.8%	0
*Escherichia coli*	5.7%	0	0
*Xanthomonas*	0	1.3%	6.3%
*Serratia* spp.	0	2.5%	0
Other Gram-negative bacilli	8.6%	7.5%	6.3%
Fungus	11.4%	15.0%	31.2%
*Aspergillus* spp.	0	8.8%	12.5%
*Mucor*	0	2.5%	12.5%
*Fusarium* spp.	2.8%	1.3%	0
*Penicillium* sp.	0	1.3%	6.3%
Uncertain^a^	8.6%	1.3%	0
Total	100%	100%	100%

^a^Uncertain fungus include fungus that could not be identified for a specific species

Table 4 shows the total susceptibility rates of isolated bacteria. In general, isolated bacteria showed the highest susceptibility (> 95%) to fluoroquinolones including levofloxacin, ofloxacin, and moxifloxacin. Gram-positive cocci had relatively high susceptibility to ceftazidime, cefuroxime, and cefazolin (84.8%, 89.5%, 87.9%, respectively), while the susceptibility to penicillin was only 59.3%. However, Gram-positive bacilli showed low susceptibility to ceftazidime, cefuroxime, and penicillin, with sensitivity rates of < 30%. The susceptibility of Gram-negative bacilli to fluoroquinolones and neomycin were > 90%, compared with 95.2% to ofloxacin.

**Table 4:** Susceptibility rate of isolated bacteria to different antibiotics in pediatric endophthalmitis

	Gram-positive cocci	Gram-positive bacilli	Gram-negative bacilli	Total specimens
Levofloxacin	96.8%	100.0%	90.0%	95.4%
	60/62	16/16	27/30	103/108
Ofloxacin	96.5%	100.0%	95.2%	96.8%
	55/57	16/16	20/21	91/94
Moxifloxacin	100%	–	–	100%
	6/6	–	–	6/6
Ceftazidime	84.8%	20.0%	65.5%	68.1%
	28/33	2/10	19/29	49/72
Tobramycin	43.5%	100.0%	73.3%	60.2%
	27/62	16/16	22/30	65/108
Neomycin	74.4%	100.0%	91.3%	83.8%
	32/43	14/14	21/23	67/80
Cefuroxime	89.5%	25.0%	40.0%	65.0%
	51/57	4/16	12/30	67/103
Cefazolin	87.9%	50.0%	19.0%	59.4%
	29/33	5/10	4/21	38/64
Vancomycin	83.3%	–	–	–
	5/6	–	–	–
Chloramphenicol	81.8%	100.0%	80.0%	84.1%
	27/33	10/10	16/20	53/63
Penicillin	59.3%	0	0	47.1%
	16/27	0/6	0/1	16/34

## Discussion

In this study, we analyzed the clinical data of 127 pediatric patients with culture-positive endophthalmitis in the southern China over a 9-year period. Gram-positive cocci comprised the majority of infections. *Streptococcus* was the most prevalent isolated pathogen, especially in the youngest age group. Fluoroquinolones including moxifloxacin, levofloxacin, and ofloxacin showed the highest antibiotic susceptibility rates. The commonly used antibiotics tobramycin and cefazolin showed relatively low antibiotic susceptibilities.

Previous studies regarding pediatric endophthalmitis are listed in Table 5. Trauma was the most prevalent etiology in our pediatric population, consistent with previous studies on pediatric endophthalmitis in the USA [12, 13]. However, the proportion of posttraumatic endophthalmitis was less than that of postoperative endophthalmitis in a predominantly adult population in the USA [21, 22]. In our study, the proportion of posttraumatic endophthalmitis (85.0%) among children was much higher than adult-dominated population reported from the same region (58.0–58.5%) [15, 23]. The higher proportion of traumatic endophthalmitis in the pediatric population could be explained by that fact that children are generally unable to either recognize or explain their symptoms after injury, thereby resulting in delayed presentation and treatment, leading to a higher incidence of endophthalmitis [24].

**Table 5:** Microbial data and susceptibility rate of isolated bacteria to different antibiotics on selected studies of pediatric endophthalmitis

Study authors and date (number of culture positive cases)	Study region	Type of endophthalmitis	The most common organism(s) (%)	Susceptibility rate of isolated bacteria to different antibiotics
Zhang et al., 2016 (*n* = 147) [14]	China	Mixed endophthalmitis	G^+^: *Staphylococcus epidermidis* (20.5%) G^−^: *Enterobacter cloacae* (2.3%) Fungi: *Fusarium species* (2.8%)	N/A
Thordsen et al., 2008 (n = 12) [13]	USA	Mixed endophthalmitis	G^+^: *S pneumoniae* (25%), *H influenzae* (25%), Fungi: *Candida spp*. (8.3%)	N/A
Al-Rashaed et al., 2006 (*n* = 49) [18]	Saudi Arabia	Exogenous endophthalmitis	Posttraumatic endophthalmitis G^+^: *Streptococcus* species (53.7%) G^−^: *Haemophillus parainfluenzae* (2.4%), *Escherichia coli* (2.4%), *Pseudomonas species* (2.4%), *Klebsiella pneumoniae* (2.4%), *Neisseria subflava* (2.4%), Fungi: *Aspergillus* species (2.4%) Postoperative endophthalmitis G^+^: *Staphylococcus epidermidis* (26.6%) G^−^: *Haemophilus parainfluenzae* (20%) Fungi: *Fusarium* species (6.7%)	N/A
Parvizi et al., 2020 (n = 8) [11]	UK	Exogenous endophthalmitis	Postoperative endophthalmitis G^+^: *Streptococcus* (28.6%), *Coagulase negative Staphylococcus* (28.6%) G^−^: *Haemophilus parainfluenzae not B* (14.3%), *Scanty Stenotrophomonas maltophilia* (14.3%)	N/A
Wu et al., 2016 (n = 10) [19]	China	Posttraumatic endophthalmitis	G^+^: *Staphylococcus epidermidis* (33.3%) Fungi: fusarium (8.3%)	N/A
Rishi et al., 2016 (*n* = 55) [3]	India	Posttraumatic endophthalmitis	G^+^: *Enterococcus faecalis* (14.5%) G^−^: *Klebsiella serratia* (10.9%) Fungi: *Aspergillus fumigatus* (3.6%)	N/A
Agarkar et al., 2016 (n = 4) [2]	India	Postoperative endophthalmitis	G^+^: *Methicillin-resistant Staphylococcus aureus* (25%) G^−^: *Acinetobacter calcoaceticus* (50%);	Vancomycin (1/2, 50%), ceftazidime (1/4, 25%), ciprofloxacin (4/4, 100%), cefotaxime (4/4, 100%)
Murugan et al., 2016 (n = 5) [6]	India	Endogenous endophthalmitis	G^+^: *Staphylococci* (20%) G^−^: *Pseudomonas aeruginosa* (20%), *Neisseria meningitides* (20%) Fungi: *Aspergillus flavus* (20%) *Candida* (20%)	N/A
Maitray et al., 2019 (*n* = 21*) [20]	India	Endogenous endophthalmitis	G^+^: *Staphylococcus epidermidis* (14.3%), *Streptococcus pyogenes* (14.3%) G^−^: *Pseudomonas* (9.5%) Fungi: *Candida albicans* (9.5%)	N/A

Mixed endophthalmitis include endophthalmitis following all causes such as trauma, surgery, systemic infection, and keratitis

Exogenous endophthalmitis include endophthalmitis following trauma and surgery. Postoperative endophthalmitis was presented following cataract surgery. G^+^ = Gram-positive organisms. G^−^ = Gram-negative organisms. * The number of cases infective by bacteria and fungi

Gram-positive cocci are the predominantly detected organisms in most endophthalmitis studies [12, 22, 25], including ours. Previous studies on pediatric traumatic endophthalmitis from Saudi Arabia and Indian demonstrated that Gram-positive bacteria accounted for 56.4–85.4% of pathogen, and *Streptococcus faecalis* and *Enterococcus* were the most common isolates, respectively [3, 18]. In our study, Gram-positive cocci dominated in patients with posttraumatic endophthalmitis, and *Streptococcus* was the most prevalent isolate. Our study also found fungal isolates accounted for 16.0% of the total, among which *Aspergillus* was the most frequently detected genus, which is consistent with our previous studies [15, 23] but lower than the values reported in studies from Turkey and India [26, 27]. The causative microorganisms can differ according to the region and environment, which may contribute to the discrepancy.

Our study found that fungi accounted for half of the isolated organisms in endogenous endophthalmitis, similar to other studies in predominantly adult populations in China and the USA [28, 29]. However, fungal infection was reported in 14.2–40% of pediatric endogenous endophthalmitis patients in India, although the sample size was small [6, 20]. The higher rate of fungal infection in our study may be related to the small number of cases and geographic differences. We found that Gram-negative bacteria are the most commonly detected pathogens in pediatric patients with bacterial endogenous endophthalmitis, which is consistent with previous analyses of adult-dominated populations in East Asia [30, 31].

In our study, four cases of endophthalmitis occurred after intraocular surgery. Bacterial infection was reported as the most common cause of endophthalmitis following pediatric intraocular surgery in previous studies [11, 32]. We found that all four cases of postoperative endophthalmitis in our series were due to bacterial infection. Al-Torbak reported that *Haemophilus influenzae* and *Streptococcus pneumoniae* were the causative organisms of pediatric endophthalmitis associated with the Ahmed glaucoma valve implant [33]. A study from the UK reported that *Streptococcus* was predominant in pediatric endophthalmitis which occurred after glaucoma surgery [11]. Similarly, *Streptococcus*, *Haemophilus influenzae,* and Coagulase-negative *Staphylococcus* were isolated in endophthalmitis after glaucoma surgery in our case series.

Two studies reported that pathogen distribution varied over time in a predominantly adult population [25, 34]. However, the shifting microbiological spectrum in pediatric patients in recent years is not adequately understood. We observed an increase in the proportion of Gram-positive bacterial infections, while those of Gram-negative bacilli and fungal infections decreased—albeit not significantly—between 2010–2014 and 2015–2018.

Most previous studies reported that coagulase-negative staphylococci were the dominant pathogens [14, 25, 35]. However, we found that *Streptococcus* was the most prevalent isolate, followed by coagulase-negative staphylococci. We also observed an obvious changing trend of isolated pathogens among different ages: streptococcus dominated in the 0–3-year-old group (40%), which was exceeded by coagulase-negative staphylococcus in the 4–12-year-old group (16.3% vs. 17.5%). In the 13–17-year-old group, the proportion of coagulase-negative staphylococcus was three times that of *Streptococcus* (18.8% vs. 6.3%). Simultaneously, the proportion of fungus increased from 11.4% to 31.2%. These shifting trends suggested that age may influence the microbiological spectrum of endophthalmitis. Previous studies demonstrated that streptococcal infection was associated with poor vision outcome in endophthalmitis [36–38]; therefore, a higher proportion of streptococcal infections in children, especially in younger children, merits clinical attention.

Antibiotic resistance is a topic of concern worldwide. Overall, bacteria showed the highest susceptibility to fluoroquinolones, consistent with our previous investigation of an adult-dominant population from 2010 to 2014, although the susceptibility rate was higher in this study [15]. Apart from fluoroquinolones, the susceptibility to other antibiotics differed among bacteria: Gram-positive cocci showed a relatively higher susceptibility to cephalosporins (~ 90%); Gram-positive bacilli showed 100% susceptibility to tobramycin, neomycin, and chloramphenicol, while Gram-negative bacilli presented higher susceptibility to neomycin (91.3%). As systemic fluoroquinolone administration is restricted in children, a combination of antibiotics is essential to achieve broader coverage against infection, before culture and susceptibility testing results become available. Since both Gram-positive cocci (84.8%) and Gram-negative bacilli (65.5%) were relatively sensitive to ceftazidime, intravenous injection of ceftazidime is recommended for the treatment of pediatric endophthalmitis. Previous studies on endophthalmitis reported that the susceptibilities of Gram-positive organisms to vancomycin were about 97.7–100% [23, 39]. In our study, the susceptibility rate of Gram-positive cocci to vancomycin was 83.3% (5/6), and the small sample size might explain the difference. As Gram-positive cocci are highly sensitive to vancomycin, intravitreal injection of vancomycin is recommended treatment for children with Gram-positive cocci infection.

The retrospective nature of this study is a limitation. This study did not analyze visual outcomes because the patients included in this study were from all over the country, hindering follow-up. In addition, younger children cannot cooperate with visual testing, leading to incomplete follow-up data. Most of the initial origin of endogenous endophthalmitis were not available in medical records either. Furthermore, we only included culture-positive cases, which could have underrepresented the overall etiological factors of pediatric endophthalmitis. Nevertheless, we provide valid data from a relatively large sample size to describe the etiology, microbiological isolates, and antibiotic sensitivities in culture-proven pediatric endophthalmitis.

In conclusion, we analyzed the clinical data of 127 culture-proven pediatric endophthalmitis in southern China. Trauma was the main etiologic factor for pediatric endophthalmitis, and *Streptococcus* was the most prevalent organism, especially in the youngest age group. Age may therefore be an influencing factor affecting the microbiological spectrum of pediatric endophthalmitis. Fluoroquinolones achieved the highest antibiotic susceptibility rate, however, commonly used antibiotics tobramycin and cefazolin showed relatively low antibiotic susceptibilities. Thus, antibiotic resistance in a pediatric population merits considerable attention.
